# Special Issue: “Soft Magnetic Materials and Their Applications”

**DOI:** 10.3390/ma17010089

**Published:** 2023-12-23

**Authors:** Shenglei Che

**Affiliations:** 1 College of Materials Science and Engineering, Zhejiang University of Technology, Hangzhou 310014, China; cheshenglei@zjut.edu.cn; 2 Research Center of Magnetic and Electronic Materials, Zhejiang University of Technology, Hangzhou 310014, China

## 1. Introduction

Soft magnetic materials normally show no magnetic properties outside of a magnetic field but can be easily magnetized and demagnetized within magnetic fields. Most soft magnetic materials have small coercive field strengths, in the same order as the Earth’s magnetic field (about 40 A/m) or lower.

Soft magnetic materials are widely used in electronics, energy conversion, information processing, and many other application scenarios. Indeed, it is safe to assume that whenever you use electricity, there are soft magnetic materials working for you. 

Recently, technological progress in soft magnetic materials has been focused on the processing of rapidly quenched amorphous and nanocrystalline materials (either in the form of ribbons or powders), as well as the improvement in magnetic properties of soft magnetic composites (SMCs) and soft ferrites to fit the requirements of high-frequency devices driven by Wide Bandgap Semiconductors (SiC/GaN). Moreover, new soft magnetic devices have also been successively designed for the rapid development of fabrication methods and new applications.

The aim of this Special Issue is to present the latest achievements in the synthesis, fundamentals, characterization, and applications of soft magnetic materials, including soft ferrites, alloys, and composites, together with their processing technology, characterization, and applications in electronics, motors, wave absorbing, sensors, communications, etc.

Seventeen papers are published in this Special Issue. For the convenience of introduction, these papers can be divided into four categories according to the contents of their studies.

## 2. Soft Ferrite Materials

Soft ferrites mainly include MnZn ferrites and NiZn ferrites. As a kind of ceramics, ferrites are normally prepared via a sintering process at 900–1450 °C. To prepare soft ferrites with small grain sizes and better high-frequency properties, Ying et al. applied cold sintering technique to sinter MnZn ferrites at around 300 °C and obtained a densified, fine-grained, high-frequency MnZn ferrite bulk [1]. The relative density could be increased to 97.2% by annealing the samples at 950 °C for 6 h. Annealing also reduced the power loss at high frequencies.

Shang et al. synthesized homogeneous MnZn ferrite fibers using the solvothermal method and used them as a reinforcing phase to prepare homogeneous-fiber-reinforced MnZn ferrite materials [2]. The best magnetic and mechanical properties were obtained at a fiber content of about 2 wt%.

Ma et al. measured the differential-mode magnetic noise in MnZn soft ferrites for magnetic shielding and analyzed and optimized the structural parameters of the shield on the differential-mode magnetic noise; some useful results for suppressing magnetic noise and breaking through the sensitivity of the magnetometer were obtained [3].

## 3. Soft Magnetic Alloys and Composites

High saturation magnetization and low coercivity FeCo soft magnetic thin films with different thicknesses were prepared via magnetron sputtering by controlling the thickness and deposition temperature in a paper by Yang et al. [4]. This FeCo thin film with high saturation magnetization and low coercivity could be an ideal candidate for high-frequency electronic devices.

Wang et al. reported a novel photodecomposition method to create a ZnO insulating layer on FeSiAl powders [5]. Combined with conventional coupling treatment processes, a thin and dense Zn-O-Si insulating layer was coated on the surface of iron powder in situ. Treating the iron powder before coating by photodecomposition led to a synergistic effect, significantly reduced the core loss, while the effective permeability only decreased slightly.

Zhu et al. studied the effect of epoxy resin (EP) on the insulated coating and pressing effect of FeSiAl magnetic powders by changing the content of EP and characterized the soft magnetic properties of the powders and SMPCs and revealed that best overall performance can be obtained when the EP content was 1 wt.% [6].

Wu et al. reported an FeSi3.5 easy-plane composite with high permeability and ultra-low loss at the MHz frequency band [7]. Through loss measurement and separation, they found that the real reason why magnetic materials do not work properly at MHz due to overheat is dramatical increase of the excess loss and the easy-plane composite can greatly reduce the excess loss. This makes the easy-plane FeSi3.5 composite become an excellent soft magnetic composite and it is possible for magnetic devices to operate properly at higher frequencies, especially at the MHz band and above.

Du et al. studied the changes of microstructure, magnetostriction properties and hardness of the Fe73Ga27−xAlx alloy and (Fe73Ga27−xAlx)99.9La0.1 alloy (x = 0, 0.5, 1.5, 2.5, 3.5, 4.5) by doping Al into the Fe73Ga27 and (Fe73Ga27)99.9La0.1 alloy, respectively [8]. The results indicated that trace La doping can improve the magnetostriction properties and deformation resistance of Fe-Ga alloy, which provides a new design idea for the Fe-Ga alloy, broadening their application in the field of practical production.

## 4. Characterization of Soft Magnetic Materials

In order to solve the problem that the relative permeability of the permalloy is missing and difficult to measure accurately in an extremely weak magnetic field (EWMF, <1 nT), Sun et al. proposed a method to measure the permeability in EWMF based on the Rayleigh model [9]. This method can obtain the relative permeability in any EWMF and avoid test errors caused by extremely weak magnetization signals.

Cheng et al. proposed a novel method of measuring the remanence of materials in a magnetic shielding cylinder, which prevents interference from the Earth’s magnetic field and reduces the measurement error [10]. This method is used to test concrete components, composite materials, and metal materials commonly applied in magnetic shielding devices and determine the materials that can be used for magnetic shielding devices with 1 nT, 10 nT, and 100 nT as residual magnetic field targets.

Wang et al. built a test system that combined temperature, stress, and electromagnetic fields along with other fields at the same time [11]. It can accurately simulate the actual complex working conditions of a motor and explore the dynamic characteristics of non-grain oriented (NGO) silicon steel. The rationality of this method was verified by checking the test results of the prototype, and the calculation accuracy of the motor model was found to be improved.

Zhang et al. investigated the magnetomechanical coupling factors (k) and damping factors (Q−1) of FeSiB amorphous ribbons annealed in air at different temperatures [12]. The k and Q−1 of FeSiB-based epoxied laminates with different stacking numbers show that a −3 dB bandwidth and Young’s modulus are expressed in terms of the magnetomechanical power efficiency for high lamination stacking.

## 5. Applications of Soft Magnetic Materials

As the core part of an electrical driving system, the electrical machine faces the extreme challenge of maintaining a high power density and high efficiency output under complex working conditions. The research and development of new soft magnetic materials has an important impact on solving the current bottlenecks of electrical machines. Li et al. studied the variation trends of magnetic properties of ultra-thin grain-oriented electrical silicon steel (GOES) under thermal–mechanical–electric–magnetic fields and explored its possible application in motors [13]. They verified that grain-oriented silicon steel has great application prospects in the drive motors (IPMs) of electric vehicles, and it is an effective means to break the bottleneck of current motor design.

High silicon steel has low loss and high mechanical strength, making it extremely suitable as a high-speed motor rotor core material. Ma et al. investigated the feasibility of using high silicon steel as the material of an interior rotor high-speed motor [14] and verified the results via theoretical analysis and experimental characterization.

Fang et al. proposed a high-performance magnetic shielding structure composed of MnZn ferrite and mu-metal film [15]. The use of the mu-metal film with a high magnetic permeability restrains the decrease in the magnetic shielding coefficient caused by the magnetic leakage between the gap of magnetic annuli. This proposed combined magnetic shielding is of great significance to further promoting the performance of atomic sensors sensitive to magnetic fields.

Ferrite magnetic shields are widely used in ultra-high-sensitivity atomic sensors because of their low noise characteristics. However, their noise level varies with temperature and affects the performance of the spin-exchange relaxation-free (SERF) co-magnetometer. Pang et al. established the thermal magnetic noise model of a ferrite magnetic shield and calculated the thermal magnetic noise of ferrite more accurately by testing the low-frequency complex permeability at different temperatures [16]. The experimental results demonstrated the effectiveness of the proposed method.

Magnetic shields are an important part of electronic equipment, ultra-sensitive atomic sensors, and in basic physics experiments. Particularly in spin-exchange relaxation-free (SERF) co-magnetometers, the magnetic shield is an important component for maintaining the SERF state. Liu et al. applied different amorphous and nanocrystalline materials as the innermost magnetic shielding layers of an SERF co-magnetometer and analyzed their magnetic noise characteristics [17]. The experimental results show that compared with an amorphous material, using a nanocrystalline material as the inner magnetic shield layer can effectively reduce the magnetic noise and improve the sensitivity and precision of the rotation measurement.

The Guest Editors would like to congratulate all the authors published in this Special Issue for the remarkable results presented in these papers. We truly believe that these works will help the research community to enhance their understanding of the present status and trends in soft magnetic materials and their applications.
